# Feasibility of Near-Infrared Spectroscopy for Monitoring Tissue Oxygenation During Uterus Transplantation and Hysterectomy

**DOI:** 10.3390/jcm14144832

**Published:** 2025-07-08

**Authors:** Jeremy Applebaum, Dan Zhao, Nawar Latif, Kathleen O’Neill

**Affiliations:** 1 Department of Obstetrics and Gynecology, Brigham and Women’s Hospital, Boston, MA 02115, USA; japplebaum1@bwh.harvard.edu; 2 Department of Obstetrics and Gynecology, University of Pennsylvania Health System, Philadelphia, PA 19104, USA; dan.zhao@uphs.upenn.edu (D.Z.); nawar.latif@pennmedicine.upenn.edu (N.L.)

**Keywords:** uterus transplant, hysterectomy, near infrared spectroscopy

## Abstract

**Background/Objective:** Thrombosis is the leading cause of graft failure and immediate hysterectomy following uterus transplantation (UTx). Currently, there is no standardized method for real-time assessment of UTx graft perfusion. This feasibility study aims to evaluate the utility of a near-infrared spectroscopy (NIRS) probe for non-invasive monitoring of local cervical tissue oxygenation (StO_2_) during UTx. As proof-of-concept for the NIRS device, cervical StO_2_ was also measured during non-donor hysterectomy and bilateral salpingo-oophorectomy to establish its capacity to reflect perfusion changes corresponding to vascular ligation. **Methods:** The ViOptix T. Ox Tissue Oximeter NIRS probe was attached to four uterine cervices during hysterectomy procedures and three separate donor cervices during UTx. Real-time StO_2_ measurements were recorded at critical surgical steps: baseline, ovarian vessel ligation, contralateral ovarian vessel ligation, uterine vessel ligation, contralateral uterine vessel ligation, and colpotomy for hysterectomy; donor internal iliac vein anastomosis to recipient external iliac vein, donor internal iliac artery anastomosis to recipient external iliac artery, contralateral donor internal iliac vein anastomosis to recipient external iliac vein, contralateral donor internal iliac artery anastomosis to recipient external iliac artery, and donor and recipient vagina anastomosis for UTx. **Results:** During hysterectomy, average StO_2_ levels sequentially decreased: 70.2% (baseline), 56.7% (ovarian vessel ligation), 62.1% (contralateral ovarian vessel ligation), 50.5% (uterine vessel ligation), 35.8% (contralateral uterine vessel ligation), and 8.5% (colpotomy). Conversely, during UTx, StO_2_ progressive increased with each anastomosis: 8.9% (internal iliac vein- external iliac vein), 27.9% (internal iliac artery-external iliac artery), 56.9% (contralateral internal iliac vein-contralateral external iliac vein), 65.9% (contralateral internal iliac artery-contralateral external iliac artery), and 65.2% (vaginal anastomosis). **Conclusions:** The inverse correlation between StO_2_ and vascular ligation during hysterectomy and the progressive rise in StO_2_ during UTx suggests that cervical tissue oximetry may serve as a non-invasive modality for monitoring uterine graft perfusion. Further studies are warranted to determine whether these devices complement current assessments of uterine graft viability and salvage thrombosed grafts.

## 1. Introduction

Uterus transplantation (UTx) is the only therapeutic option that enables women with absolute uterine factor infertility to achieve pregnancy [1]. Stable vascular perfusion is critical for the viability of the uterine allograft, yet approximately 20% of UTx result in emergent postoperative hysterectomy due to thrombosis or graft hypoperfusion [2,3,4]. Compromised graft perfusion may relate to the complexity or constriction of donor-recipient vascular anastomoses or underlying donor atherosclerosis [2,3,5]. The development and application of technologies capable of accurately detecting and facilitating timely intervention for compromised graft perfusion are crucial for advancing the field of UTx.

Currently described postoperative monitoring techniques for graft perfusion include transabdominal or transvaginal Doppler ultrasonography and the Cook-Swartz Doppler flow probe (Cook-Swartz Doppler Probe; CooperSurgical) [2,6,7]. The Cook-Swartz probe consists of a 20-megahertz crystal ultrasound transducer within a silicon cuff. It can be affixed around an arterial supply to the donor uterus, connected to stress retention tabs, and connected to an external terminal for monitoring [6]. While these modalities have been used to monitor blood flow postoperatively following reconstructive vascular surgery, each has notable limitations [2,3]. Doppler ultrasonography is limited by documented false-negative results in thrombosed grafts [3]. The Cook-Swartz probe is invasive and cannot detect venous thrombosis [2,3]. Angiography can confirm suspected thrombosis once it has already occurred, but rarely allows for graft salvageability [3,8]. Angiography also imposes a radiographic contrast load on patients at risk for acute kidney injury, given recent induction with nephrotoxic immunosuppressants [9]. Importantly, these methodologies are all measures of peripheral blood flow and oxygen saturation (SpO_2_), which may not accurately reflect local tissue oxygenation (StO_2_) due to differences in oxygen delivery and consumption [10].

Near infrared spectroscopy (NIRS) represents a promising, non-invasive technology for real-time monitoring of UTx cervical StO_2_. NIRS probes have improved thrombosis detection and graft salvage rates in breast, extremity, truncal, and head and neck free flaps within the plastic surgery literature [11,12,13,14,15]. This study aims to evaluate the feasibility of cervical intra- and post-operative StO_2_ monitoring with an NIRS probe device during UTx.

## 2. Materials and Methods

**Figure 1:**
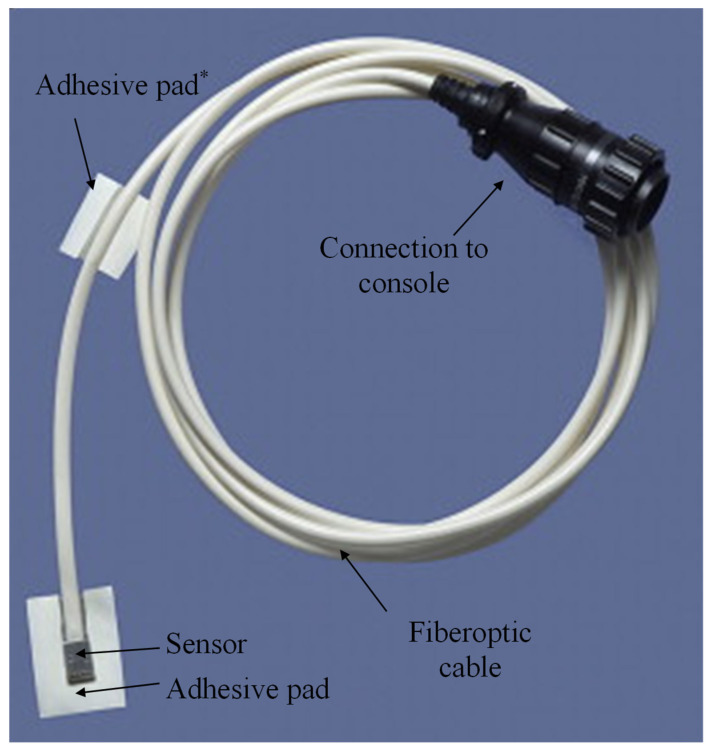
The ViOptix probe with adhesive pad attached to the fiberoptic cable. * Not used in this study.

**Figure 2:**
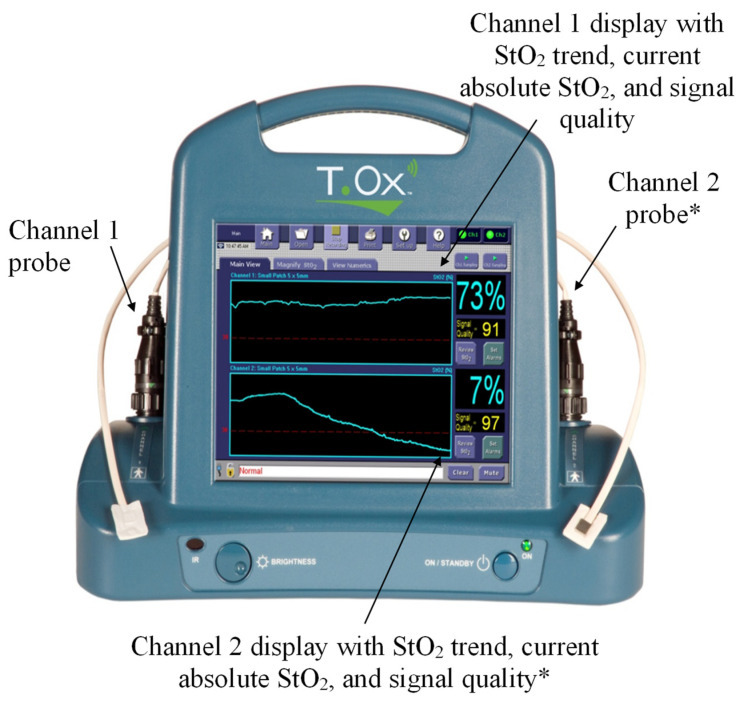
ViOptix console alongside two-channel probes with a sample display of StO_2_ measurements. * Not used in this study.

The device being studied is the ViOptix T. Ox Tissue Oximeter (ViOptix, Inc., Fremont, CA, USA) and is patent-protected, Food and Drug Administration-approved for tissue oxygenation monitoring [16]. It consists of a five-millimeter by five-millimeter flat sensor with two NIRS lasers that penetrate tissue up to one centimeter deep and four photoelectric diodes that detect reflected light (Figure 1). Measurements of infrared light scattering and absorption due to local tissue hemoglobin and deoxyhemoglobin concentrations are used to calculate StO_2_. A fiberoptic cable connects the sensor to a console that displays continuously recorded StO_2_ measurements (Figure 2) [11,12]. The console also displays an indicator of signal quality, a measure of the consistency of StO_2_ readings between the four diodes. A signal quality of at least 80% is considered satisfactory for the reliability of StO_2_ readings for clinical purposes. While the ViOptix console allows for dual monitoring of two probes, only one channel was used in this study.

As proof of concept, the ViOptix device was used to assess cervical StO_2_ measurements during hysterectomy. Patients undergoing a total abdominal hysterectomy and bilateral salpingo-oophorectomy (TAH-BSO) with a gynecologic oncologist at the Hospital of the University of Pennsylvania (Penn Medicine) were enrolled from August 2018 to February 2019. At the beginning of the surgery, vaginal retractors were placed and the ViOptix device was sutured to the cervix. The cable was passed through the vagina and connected to the console. StO_2_ measurements at key surgery steps of the TAH-BSO (ovarian vessel ligation, contralateral ovarian vessel ligation, uterine vessel ligation, contralateral uterine vessel ligation, and colpotomy) were recorded. Once the specimen was freed, the sensor was removed from the cervix and the cable removed via the vagina.

Following the pilot study, the ViOptix device was used for research purposes in subjects undergoing UTx. All patients who underwent UTx at Penn Medicine from November 2018 to February 2020 were enrolled. Patients were recruited through Penn Medicine’s Uterine Transplantation for Uterine Factor Infertility (UNTIL) trial [17].

The ViOptix device was used for both intraoperative and postoperative StO_2_ monitoring in UTx. During backbench preparation of the donor uterine grafts, the ViOptix sensor was sutured to the left lateral aspect of the donor cervix. Once the recipient’s pelvic vasculature was surgically exposed and colpotomy was made, the graft was brought to the operative field, and the ViOptix cable was fed through the colpotomy and connected to an external console. StO_2_ measurements were then continuously recorded at five-second intervals for the duration of surgery. StO_2_ measurements at key surgical steps of the UTx (donor internal iliac vein anastomosis to recipient external iliac vein, donor internal iliac artery anastomosis to recipient external iliac artery, contralateral donor internal iliac vein anastomosis to recipient external iliac vein, contralateral donor internal iliac artery anastomosis to recipient external iliac artery, and donor and recipient vagina anastomosis) were recorded. Cervical StO_2_ measurements were also recorded for up to three days postoperatively. The ViOptix device was removed during a scheduled, routine post-UTx exam and cervical biopsies under anesthesia.

The criteria studied in both the UTx and TAH-BSO patients were the absolute StO_2_ value, the amount of its change (ΔStO_2_), and the rate of its change (ΔStO_2_/Δtime). Given that there is no published use of the ViOptix device in gynecologic surgery, surgical reexploration of a UTx was not performed in this study due to isolated StO_2_ values or StO_2_ trends.

Study participants provided written consent. This study was approved by the Institutional Review Board of the University of Pennsylvania (UNTIL #827853, ViOptix #829582).

## 3. Results

### 3.1. Participant Demographics

Four patients undergoing a TAH-BSO were enrolled. All TAH-BSO patients underwent surgery for suspected gynecologic malignancy, with pathology-confirmed malignancy in all but one patient. Only one patient (H2) underwent neoadjuvant chemotherapy prior to surgery.

All three patients who underwent a UTx at Penn Medicine during the study timeframe were enrolled. All UTx patients had a diagnosis of Mayer–Rokitansky–Küster–Hauser syndrome or congenital absence of the uterus. Participant demographic features are listed in Table 1. Table 1 also displays the pregnancy outcomes of Penn Medicine’s UNTIL trial.

### 3.2. Hysterectomy and Bilateral Salpingo-Oophorectomy Intraoperative Monitoring

**Figure 3:**
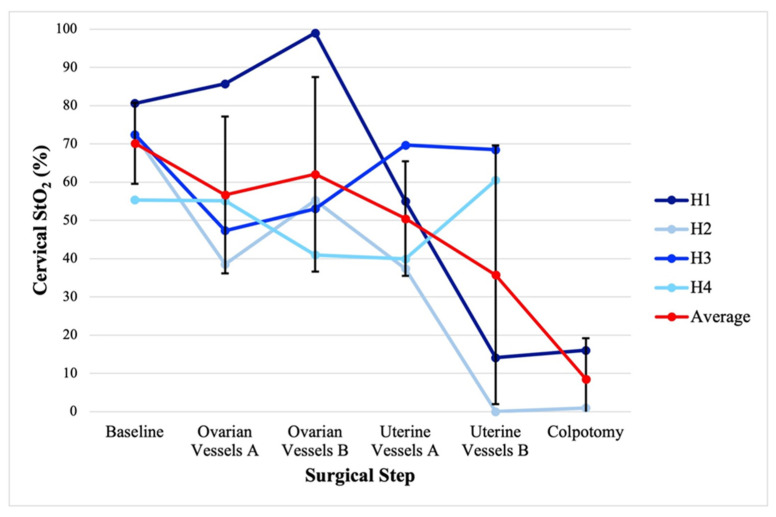
Cervical StO_2_ by the key steps of hysterectomy and bilateral salpingo-oophorectomy. A and B represent laterality. Ovarian vessels with regard to ligation of the infundibulopelvic ligament. Uterine vessels with regard to ligation of the uterine artery and uterine vein. Error bars represent standard deviation.

A combined 2760 unique StO_2_ measurements were gathered for the four TAH-BSO patients over a time range of 1:09:43–1:52:14 (hours:minutes:seconds). Figure 3 displays the cervical StO_2_ for each TAH-BSO patient and the average by key surgical step. The average (standard deviation) cervical StO_2_ for the sequential steps of baseline, ovarian vessel ligation, contralateral ovarian vessel ligation, uterine vessel ligation, contralateral uterine vessel ligation, and colpotomy was 70.2 (10.6)%, 56.7 (20.5)%, 62.1 (25.4)%, 50.5 (15.0)%, 35.8 (33.8)%, and 8.5 (10.6)%, respectively.

### 3.3. Uterus Transplantation Intraoperative Monitoring

**Figure 4:**
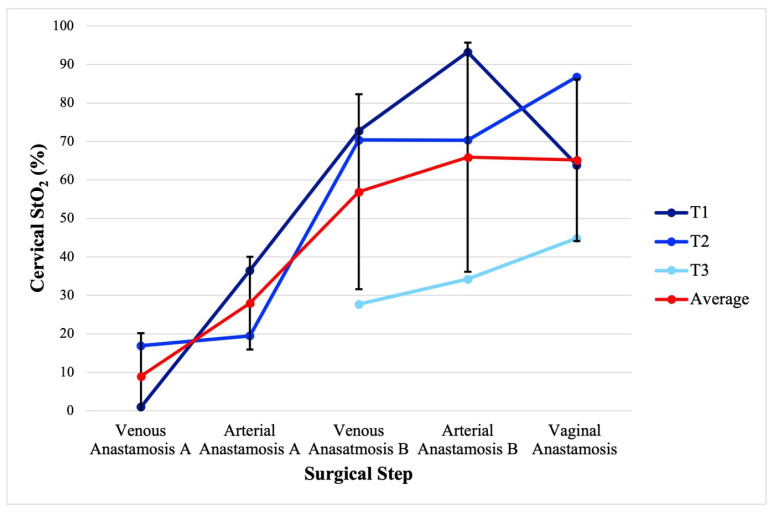
Cervical StO_2_ by the key steps of uterus transplantation. A and B represent laterality. Venous anastomosis refers to the donor internal iliac vein anastomosis to the recipient external iliac vein. Arterial anastomosis refers to the donor internal iliac artery anastomosis to the recipient external iliac artery. Error bars represent standard deviation.

Figure 4 displays the cervical StO_2_ for each UTx patient and the average by key surgical steps. The average (standard deviation) cervical StO_2_ for the sequential steps of uterus transplantation of recipient external iliac vein to donor internal iliac vein anastomosis, recipient external iliac artery to donor internal artery anastomosis, contralateral external iliac vein- internal iliac vein anastomosis, contralateral external iliac artery-internal iliac artery anastomosis, and vaginal anastomosis was 8.9 (11.3)%, 27.9 (12.0)%, 56.9 (25.3)%, 65.9 (29.8)%, and 65.2 (21.0)%, respectively.

### 3.4. Postoperative Monitoring of Uterus Allograft

A combined 90,746 unique StO_2_ measurements were gathered for the three UTx patients over a time range of 0:08:56:34–3:04:04:51 (days:hours:minutes:seconds), including both intraoperative time and postoperative monitoring.

**Figure 5:**
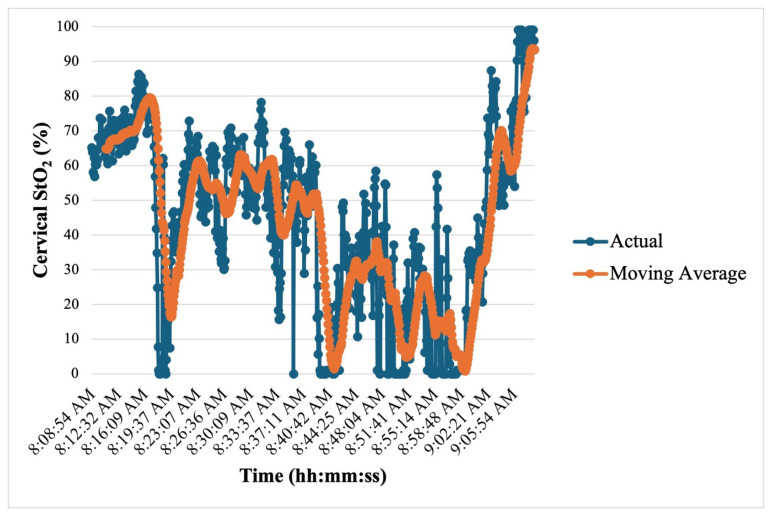
Abrupt decline in patient T1 cervical StO_2_ on postoperative day 1. Actual values are depicted in blue. A 10-interval moving average is depicted in orange.

Notably, in patient T1, approximately 19 h postoperatively, an abrupt decline in absolute cervical StO_2_ from 86.3% to 7.7% was observed, followed by eventual spontaneous rebound to StO_2_ measurements greater than 90% approximately 60 min later (Figure 5). Given that no clinical decisions were made using ViOptix, and no interventions were performed. Routine Doppler ultrasonography three hours later demonstrated adequate bilateral uterine arterial flow. On postoperative day (POD) six, the patient underwent computed tomography of her abdomen and pelvis for an unrelated indication, and an incidental occlusion was noted along the right external iliac artery. Magnetic resonance angiography confirmed an occluded graft between the recipient right external iliac artery and the donor right internal iliac artery, with reconstitution at the level of the right common femoral artery via the inferior epigastric artery. Right venous and left arterial and venous vascular patency was noted. Vascular surgery was consulted. Given the lack of symptoms and persistent radiographic uterine enhancement, likely through collaterals or contralateral supply, the decision was made to leave the graft in-situ. The patient was initiated on therapeutic anticoagulation, which was continued for eight weeks postoperatively.

The ViOptix probes were removed on POD three or four during scheduled exams under anesthesia. There was poor application of the ViOptix sensor to the cervix in all three cases at the time of removal.

## 4. Discussion

This feasibility study demonstrates a relative increase and decrease in human cervical StO_2_ throughout the surgical steps of UTx and TAH-BSO, respectively, suggesting that the ViOptix device may be used to monitor the perfusion dynamics of the human cervix. Given the novelty of NIRS monitoring in uterus transplantation, there are no established recommendations for graft intervention based solely on StO_2_ values.

One case of an abrupt postoperative StO_2_ decline occurred in the setting of a graft thrombosis incidentally detected days after the StO_2_ drop off. However, the suggestion of a causal relationship is unwarranted, as arterial ultrasonographic Doppler flow remained normal, and postoperative ViOptix signal quality was intermittently poor. These confounding factors preclude attribution of the StO_2_ decline to the thrombosis event. There are several plausible reasons why the ViOptix device may not function as effectively on the human cervix as has been reported in the free flap literature [11,12,13,14]. These include differences in surface topography, with the cervix having a curved, mucosal surface, unlike the relatively flat and keratinized surface of skin. This may limit consistent contact and probe adherence. Cervical tissue is also composed primarily of smooth muscle and both squamous and glandular epithelium, in contrast to the collagen-rich dermis of skin. These tissue-specific compositions likely have different optical properties, which could affect signal interpretation. In addition, the vaginal environment, with distinct lighting, humidity, and temperature, may influence sensor performance. Cervical inflammation or physiologic discharge can further alter optical properties and probe-surface interaction. The cervix is also subject to motion from patient activity or changes in bladder and bowel filling, increasing the risk of signal artifact.

StO_2_ has been described as an adjunct to clinical examination in monitoring non-gynecologic grafts. The ViOptix device and other NIRS-based point probes have demonstrated efficacy in both detecting and preventing graft thrombosis in various free flap surgeries [11,12,13,14]. Given that vascular thrombosis is the leading cause of immediate graft failure in UTx, NIRS may have potential application as a non-invasive means of postoperative UTx graft monitoring [2,18].

There is limited data on the application of NIRS probes in the surgery of the human uterus and cervix. Prior work has used NIRS probes to demonstrate that different optical properties of the human cervix occur throughout the menstrual cycle, pregnancy, obstetric cervical dilation, and in the presence of dysplasia [19,20,21,22]. Uterine corpus StO_2_ mapping as measured through a spectroscopy monitor affixed to a laparoscope has also been performed on UTx in sheep and rabbit models [23].

There are several potential advantages of NIRS probes for UTx graft monitoring. NIRS probes provide real-time, continuous monitoring, unlike intermittent ultrasonographic or clinical assessments. This may allow for decreased resource allocation for postoperative graft care [11]. These devices are also non-invasive and can be both affixed and removed from the cervix via a speculum examination. NIRS probes also provide information on oxygenation in the tissue of interest as opposed to its vascular supply, which may differ due to oxygen dissociation from local temperature or pH [10,24].

This study is not without limitations. The study was limited to a small pilot cohort of seven patients—three who underwent UTx and four who underwent hysterectomy. This is partly attributable to the very low incidence of UTx, with many clinical and surgical aspects remaining investigational. The primary aim was to assess the feasibility of using NIRS to monitor local StO_2_ of the human cervix. Consequently, the sample size limits the scope of the analyses, statistical power, and generalizability. The use of a small pilot cohort also precludes assessment of whether ViOptix can reliably detect graft thrombosis or improve salvage rates with UTx. Therefore, these findings should be considered exploratory, and we do not advocate for routine application of NIRS probes for UTx monitoring without more comprehensive investigation. Device cost may also be a barrier to routine application, although this may be offset by potential reduction in costs associated with graft loss and reoperation [11]. Notably, while UTx anastomotic steps were consistent in this study, alternative vascular anastomotic approaches in UTx could impact StO_2_ patterns. Variability may also exist between deceased and living donor uterus allografts. Though a downtrend in cervical StO_2_ was noted with the subsequent steps of a hysterectomy, most of these patients had a gynecologic malignancy, which may result in aberrant cervical perfusion either due to the malignancy itself or prior antineoplastic treatments. While cervical application allows for NIRS probes to be non-invasively applied, it is unclear if cervical StO_2_ is an adequate proxy for the entire uterine perfusion, especially considering a limited depth of laser penetration. The flat, paddle-shaped design of the ViOptix probe may also pose challenges in achieving full contact with the naturally curved cervix, especially given postoperative edema, inflammation, or ischemia-reperfusion effects. Future adaptations to the probe’s shape and adherence mechanism are needed to improve conformity to the convex anatomy of the ectocervix and maintain reliable contact. A clearer understanding of the cervix’s light-scattering and absorption properties is also necessary to guide proper device calibration and optimization for this unique application.

More important than the development of novel technology to monitor graft perfusion, however, is the establishment of criteria to prompt clinical intervention. Identification of isolated StO_2_ levels or a decreasing StO_2_ trend that may prompt more intensive monitoring or surgical intervention is needed. Correlates from the breast, head, and neck, as well as the truncal free flap literature, demonstrate that StO_2_ values between 30–40% and a StO_2_ decrease of 15–20% within one hour are predictive of flap failure [12,13,25]. However, these thresholds should not be applied to UTx without further validation. Unlike free flaps, which involve microsurgical arterial and venous reconstitution, vascular compromise of the uterus and cervix after UTx occurs in deeper, larger-caliber vessels. Additionally, oxygen consumption and, therefore, relative hemoglobin and deoxyhemoglobin concentrations may differ significantly between a uterus allograft and a free flap. Larger studies are needed to identify StO_2_ criteria through NIRS probes that predict UTx failure.

While we have demonstrated that a NIRS probe for intra- and post-operative StO_2_ monitoring during UTx may be feasible, more robust studies are needed to expand these findings and assess clinical utility. Additional studies are needed to determine whether these devices may improve the detection of thrombotic graft compromise and improve salvage rates.

## 5. Conclusions

The ViOptix NIRS probe applied to the cervix at the time of hysterectomy and uterus transplantation demonstrated a relative decrease and increase in StO_2_, respectively. The development and refinement of technologies to non-invasively monitor uterine perfusion may be an important factor in optimizing UTx outcomes.

## Figures and Tables

**Table 1:** Uterus transplantation and hysterectomy participant demographics.

**UTx**	**Patient Identifier**	**Age at Surgery (y)**	**BMI (kg/m^2^)**	**Race**	**Donor Allograft Status**	**Subsequent** **Pregnancy Outcomes**
	T1	32	19	White	29-year-old deceased donor, three prior vaginal deliveries	1 live-born infant, cesarean hysterectomy
	T2	26	32	White	35-year-old deceased donor, two prior vaginal deliveries	2 live-born infants: cesarean section, followed by cesarean hysterectomy
	T3	32	21	White	41-year-old living donor, one prior vaginal and one cesarean section	2 live-born infants, cesarean section, followed by cesarean hysterectomy
**TAH-BSO**	**Patient Identifier**	**Age at Surgery (y)**	**BMI (kg/m^2^)**	**Race**	**Pathology**	**__**
	H1	62	28	Black	Uterine carcinosarcoma	
	H2	62	42	White	Ovarian serous carcinoma	
	H3	71	37	Black	Ovarian mucinous cystadenoma	
	H4	56	30	Black	Endometrial endometrioid carcinoma	

## Data Availability

The data presented in this study are available upon reasonable request from the corresponding author due to privacy concerns, given the small number of patients in this study and the ease of personal identification.
